# Lessons Learned About Digital Health Tool Acceptability Among Rural Older Adults: Systematic Review Guided by the Technology Acceptance Model

**DOI:** 10.2196/70012

**Published:** 2026-02-04

**Authors:** Zachary M Siegel, Ellie Quinkert, Jiya Pai, Corinne H Miller, Marquita W Lewis

**Affiliations:** 1 University of Kentucky Lexington, KY United States; 2 Case Western Reserve University Cleveland United States; 3 Northwestern University Chicago, IL United States; 4 Department of Medical Social Sciences Northwestern University Chicago, IL United States

**Keywords:** health services, older adult, rural health, digital health, social determinants of health

## Abstract

**Background:**

Digital health tools are increasingly vital in rural health care due to widespread hospital closures and the rapid adoption of telehealth during the COVID-19 pandemic. Rural older adults, a uniquely vulnerable population, face barriers to accessing these tools due to rurality and usability challenges. Although a growing body of literature examines the acceptability and usability of digital tools among rural older adults, no study has synthesized this research to establish best practices.

**Objective:**

This study aims to review existing literature on digital health tools for rural older adults, highlighting key lessons learned about their acceptability and identifying strategies to improve usability for this population.

**Methods:**

Following the PRISMA (Preferred Reporting Items for Systematic Reviews and Meta-Analyses) guidelines, this study reviewed literature that investigated the role of digital health tools on the health outcomes of rural older adults (ie, at least 60 years old). The literature was retrieved from 5 electronic databases through June 2023. This study and all reviewed literature were conducted in the United States. Guided by a systematic process, 2 reviewers assessed relevant articles for eligibility, analyzed data, and extracted relevant content. The extracted findings were organized according to the evidence-based technology acceptance model, which assesses the acceptability of a technology by its usefulness, ease of use, and intention to use.

**Results:**

The preliminary title review produced 7728 results, and 38 eligible manuscripts were included in the final review. Studies included both rural older adults and providers of rural older adults as participants. Digital health tools included, but were not limited to, videoconferencing, phone calls, telehealth monitoring, telemedicine appointments, and computer-based interventions. Findings on the usefulness of digital health tools by rural older adults were mixed. While digital health tools were useful for overcoming barriers to accessing care, these tools were less useful for rural older adults with limited digital literacy. Additionally, some studies described that the technology was easy but difficult to use when faced with environmental barriers, equipment issues, and discomfort with the technology. Rural older adults often reported an intention to use the technology after the study. Yet, on a few occasions, participants who preferred in-person care visits or did not have buy-in on the technology reported no intention to use the technology again.

**Conclusions:**

Our review highlights that rural older adults and their providers generally view digital health tools as acceptable for delivering care and, in some cases, as a viable alternative to in-person clinic visits. While certain barriers impacted the acceptance of these tools among rural older adults, many of these challenges were not directly linked to their age or rural location; thus, they are potentially applicable to urban older adults.

**Trial Registration:**

PROSPERO CRD42021287924; https://www.crd.york.ac.uk/PROSPERO/view/CRD42021287924

## Introduction

Digital health tools facilitate communication between patients and health care providers and offer access to resources. These tools encompass a range of technologies, including mobile health apps, electronic health records, wearable devices, and telehealth services. Social distancing mandates related to COVID-19 facilitated increased funding to support improved access to broadband internet and the rapid uptake of digital health tools [1]. To increase digital tool access and use by rural residents, in the spring of 2022, the US Department of Health and Human Services announced a US $16.3 million expansion to telehealth care in the Title X Family Planning Program [2]. Thus, rural health care professionals and systems were able to integrate digital tool uptake in their care rapidly [3]. Telehealth uptake in health clinics and hospitals increased by 154% in March 2020 compared with March 2019 [4]. For many rural patients, digital health tools are an essential component of their health care management and will likely remain important for timely and continuous rural care coordination [1].

Rural older adults represent a vulnerable population at the intersection of aging and rural residency, facing well-documented yet preventable challenges in accessing health care [5]. Rural residents are rapidly aging in place. For instance, 25% of older adults live in a rural or small town, and this is expected to rise to 33% by 2030 [6]. Additionally, for many rural older adults, care management is complex, confusing, and further challenged by coordination between distant health care facilities [7]. Since 2010, over 160 rural hospitals have permanently closed their doors, reducing access to inpatient care, which is critical for improving rural community health [8]. Therefore, aging rural populations will increasingly experience limited access to specialty care and poorer health outcomes [9].

Digital health tools can potentially overcome care coordination challenges for rural older adults. Once rural older adults engage with digital health tools, they often find their experience satisfactory and, at times, comparable to in-person visits [10]. Rural older adults evaluated web-based consultations conducted by service providers with high efficiency and satisfaction scores [11]. Once older adults understand the technology, they often find it an acceptable mode of care when punctuated by in-person visits.

Despite high levels of satisfaction with digital tools by rural older adults, compared with urban older adults, this vulnerable population has reduced telehealth use [12,13]. Also, although rural residents are willing to adopt digital health tools [14,15], studies show that rural older adults report slower telehealth uptake than younger rural adults [16,17]. This is partially due to barriers that make using digital health tools difficult for rural older adults. Some of these barriers include technical literacy, lack of technical support, cost, ownership of technology, and visual acuity [13]. In a systematic review including rural adults aged 55 years and older who have used telehealth, older adults reported a willingness to learn how to use various digital tools, but 30% felt too inexperienced with technology to use them [18]. Similarly, in a sample of Medicare enrollees, rural cancer survivors had a significantly lower predicted probability of internet use for patient-provider communication when compared with urban cancer survivors with Medicare (28% vs 46%) [19]. Importantly, not all rural older adults will find digital health tools to be a favorable health care management tool. Yet, funding to increase broadband access and the threat of widening rural medical deserts will facilitate continued telehealth uptake of digital tools by health care systems, thereby reinforcing the increased uptake of digital health tools by rural older adults. Increasing the acceptability of digital health tools and reducing barriers to their uptake for rural older adults are essential for providing health care to rural older adults.

Given the rapid acceleration of digital health tools by rural health care providers, rural older adults find digital health tools helpful. Still, rural older adults have reduced uptake of these technologies compared with both urban older adults and younger rural adults. With the increasing use of digital health tools, understanding their acceptability among rural older adults is crucial for ensuring this vulnerable population stays engaged in their care management and coordination as reliance on these tools continues to grow. Details about the rural older adults’ digital health tool acceptability and usage can inform tool intervention design, implementation, and evaluation. Existing research summarizes the effectiveness of services such as telehealth among older adults, but strategies to improve rural older adults’ usage of digital health tools are limited [18]. Therefore, this study will systematically review the existing literature in the United States on rural adults’ acceptability of digital health tools and assess lessons learned on digital health tool usage among rural older adults.

## Methods

### Study Design

The study was analyzed and reported in accordance with the Cochrane systematic review guidelines and the PRISMA (Preferred Reporting Items for Systematic Reviews and Meta-Analyses) 2020 guidelines (Multimedia Appendix 1) [20,21].

### Search Strategy and Data Sources/Protocol Registration

A trained librarian conducted searches in MEDLINE (Ovid), Cochrane Database of Systematic Reviews (Wiley), Embase (Elsevier), CINAHL (EBSCO), and PsycINFO (EBSCO) databases. This search included articles published in English through June 5, 2023. Keywords and subject headings related to the following topics were used to identify possible articles: rural residents, older adults, the use of technology-enhanced tools to navigate telehealth, and acceptability. See the supplementary materials for the complete search strategy. Following, we searched the references of eligible articles for additional relevant articles. The protocol was registered post hoc in the International Prospective Register of Systematic Reviews (PROSPERO CRD42021287924).

All articles eligible for data extraction underwent title and abstract review, and full-text review by a pair of reviewers. Reviewers independently assessed the articles based on the eligibility criteria (see below) using standardized procedures in the systematic review software Covidence (Covidence systematic review software, Melbourne, Victoria, Australia). A pair of reviewers discussed and resolved conflicts in weekly meetings. Each article was assessed for quality by 2 reviewers. Extracted content and Quality Assessments were reported using Microsoft Excel and Covidence.

### Eligibility Criteria

We included research articles of investigations conducted in the United States that assessed a digital health tool’s ability to connect patients with providers, where at least 25% of the sample population identified as rural, and at least 25% of the sample identified as at least 60 years old. Articles were excluded if they were a review (eg, systematic or scoping), withdrawn, a conference proceeding, an abstract, or a dissertation. We also excluded articles published before 1999, as we deemed that the technology or lessons learned from the technology over 25 years ago were antiquated.

### Data Extraction

Paired reviewers conducted consensus meetings to agree upon the rationale for data extraction content and synthesize the results. Table 1 displays the extraction content. In short, paired reviewers reported each article’s title, first author, and year of publication. The outcome variables collected from each study included the participants’ age (average or mean), study design, and type of digital health tool technology (eg, videoconference or wearables). Following the technology acceptance model (TAM), we extracted data related to the core TAM domains: perceived usefulness, perceived ease of use, and intention to use [22]. The perceived usefulness domain describes how much technology improves a patient’s performance. Perceived ease of use is the effort required to use the technology. Last, intention to use refers to a patient’s willingness to use the technology. Given that we aim to synthesize acceptability and lessons learned, our analysis did not assess the effect of the outcomes. This systematic review did not need an exploration of heterogeneity or a sensitivity analysis.

**Table 1:** Summary of findings.

Study	Population characteristics		Study design	Technology	Usefulness	Ease of use	Intention to use
	Age	Rural					
							
Anderson et al [23]	Mean age 61 years	Community-based outpatient clinics in rural Southeast Texas	Qualitative	Videoconferencing	It was accessible. Videoconferencing was convenient and reduced transportation issues.	Participants reported that there was too much information covered in the 2 self-management classes and that 2.5 hours was too long for a single videoconferencing session. Clinicians reported that they had no time to assist when technology problems occurred. Clinicians reported that there was only limited clinic space to hold videoconferencing sessions.	Not applicable.
Barton et al [24]	28.5% of the sample is 60 years or older	67.3% of the sample is from rural Colorado	Cross-sectional	Phone call, videoconference	Providers reported that ease of access to patient records, scheduling follow-up visits, and timely follow-ups were all better accomplished during digital visits than office visits (2-4.5× higher, all P<.001).	Not applicable.	Patients who did not engage in telehealth listed “preference to seeing the provider in person” and “telehealth not being an option” as reasons.
Bernacchi et al [25]	Mean age 60.2 years	Rural dwelling residents from Southeast US	Mixed methods	Videoconferencing	Access to an oncology nurse during COVID-19 increased rural cancer patients’ access to care, information, and education. Participants gave unfavorable scores to questions that asked about the use of the technology. For example, the lowest scoring items were “my health is better than it was before I used the technology” (—X = 3, SD = 0.89).	Participants struggled with connecting to appointments due to a lack of equipment or discomfort with digital technology. Three participants with insufficient broadband used nearby telehealth satellite sites at local clinics or hospitals. Participants often relied on family members for connection due to limited internet experience or poor broadband signals.	Participants were committed to overcoming barriers in order to speak with their nurse.
Bonsignore et al [26]	Mean age 72 years	Rural counties in Western North Carolina	Mixed methods	Telemonitoring	TapCloud increased patient-provider relationship, accessibility to clinicians, increased response times, improved efficiency, and made medications more accessible. Accessibility to a provider increased comfort, along with communication and preemptive management of problems.	TapCloud’s facilitation of direct, efficient contact with patients made it easy to use. Patients and caregivers were particularly enthusiastic about how easy medication refills are with TapCloud. Patients and caregivers find the TapCloud application intuitive, easy to use, and not time-intensive.	Once coached on using the application, patients readily adopted the technology and often felt a sense of accomplishment in doing so.
Browning et al [27]	Mean age 81 years	Residents of rural Southwest Virginia	Retrospective quality improvement case series	Phone-based telehealth monitoring	Participants felt nurses had better accessibility to vitals (88.9%). 90% felt a better connection with their doctor.	77.8% found telehealth easy to use.	Not applicable.
Collie et al [28]	Mean age 60.7 (SD 9.24) years	Resident of Intermountain region of North-eastern California	Pretest-posttest	Videoconferencing	Not applicable.	Participants adapted quickly to videoconferencing and reported no communication difficulties. The facilitator faced challenges due to time lags and poor lighting.	All said they would recommend it to other women with breast cancer.
Cummings et al [29]	Mean age 52.8 (SD 16.2) years	Rural counties in eastern North Carolina	Descriptive	Nonmydriatic retinal imaging telemedicine system	85% of images were “good or fair” when examined by a retinal specialist. Retinal specialists reported “very certain or certain” in 84% of diagnoses.	96.3% of the participants were “very comfortable” or “comfortable” with the portable camera.	Not applicable.
DeHart et al [30]	41.2% of the sample is 55 years or older	Rural resident from South-Eastern State	Mixed methods	Web and mobile-based telehealth with remote monitoring	The application effects were seen through the increased coordination across agencies, technology fit with intraoffice demands, growing practice through engagement, flexible delivery, reduced provider travel, geographic access, reduced patient transport, saving time/visits for patients, reduced costs, and fewer challenges to sustainability.	Telehealth had organizational technology and space challenges. Patient skills and comfort were a challenge. Provider knowledge and skills were a challenge. Rural patients struggled with technology access, often due to a lack of broadband technology in homes, but also due to technology skills for aging populations and those with less education.	The most common patient-level challenge reported by providers was a lack of buy-in to use the service consistently.
Demaerschalk et al [31]	Mean age 66.3 (SD 13.5) years	Patients at rural medical centers, more than 185 miles from a primary stroke center	Randomized control trial	Telestroke	Results indicate effectiveness in the organization and structure of stroke telemedicine networks for extending stroke care in rural communities.	Technical problems were noted in 20 of 27 (74%) of telemedicine consultations and in 0 of 27 telephone consultations. However, none of the issues kept the patient from being cared for.	Not applicable.
Depatie and Bigbee [26]	Senior living facility serving adults aged 60 years or older	Rural northern California senior centers	Descriptive mixed methods	Mobile health technology	67% felt it was moderately important for the technology to monitor their health, but do not feel comfortable sharing with their provider. 53% felt it was somewhat or extremely important to monitor their health and were comfortable sharing information with their provider. 46% felt it was somewhat or extremely important to monitor their health as well as share information over the internet with their health care provider. 44% indicated it was moderately, somewhat, or extremely important to use mobile health technology in combination with in-home nurse visits. 40% felt it was somewhat or extremely important to connect patient education and support groups online. 77% felt health technology had a clear benefit on their health.	Participants’ comfort level with using email or the Internet to communicate with a health care provider was evenly split.	Responses to interest in incorporating technology into daily life for health tracking and communication with a health care professional indicated that 33% had no interest, 23% were somewhat interested, and 13% indicated that they already used this technology.
DeVido et al [32]	Mean age 54 (SD 19.4) years	Providers at a rural population-serving hospital	Case report	Telepsychiatry	Telepsychiatry is capable of responding to many consultation questions in a hospital setting. Cognitive test items were collected reliably using a camera function that interpreted physical images. Telemedicine gave valuable clinical data regarding psychotic symptoms. Telepsychiatry was applicable in accessing a diverse range of patients. Communication between the resource nurse and referring physician was aided through telemedicine.	Technological challenges included getting the cameras and software to operate reliably. An ongoing challenge was the integrity of the image and audio. Audio feed was compromised by Wi-Fi connectivity problems.	Not applicable.
Donahue et al [33]	Mean age 57.9 (SD 12.4) years	Rural North Carolina county	Cohort	Phone-based digital health care	84% of participants set a goal and reached at least one goal due to digital health technology.	Not applicable.	Providers expressed interest in continuing the phone coaching program with their participants, if resources were available. 96% would recommend phone coaching to others. 58% of participants remained engaged with phone coaching over the 12-month period (missed fewer than 3 consecutive monthly calls), 17% were less engaged (received at least one call), and 25% were not engaged.
Finley et al [34]	82% of the sample is 65 years or older	Three rural outpatient telehealth clinics, or one urban outpatient clinic in Arizona	Mixed methods	Telecardiology	Patients perceived service as competent and began to see benefits in accessibility, reduced transportation, and general timesaving.	Not applicable.	When compared with the telehealth group, in-person participants more frequently expressed concerns about technology, about care not being face-to-face, and made more negative statements about quality. Rural participants held the most positive attitudes toward telehealth, while suburban participants held the least positive attitudes toward telehealth.
Geller et al [35]	54.6% of the sample is 61 years or older	Patients and their providers from rural practices in Vermont	Quasi-experimental	Computer-based interactive interventions	Preliminary evidence that the technology could educate patients about screening. Technology promoted patient-provider discussion, provider recommendations, and positive patient intentions to get a screening.	Digitally everyone found the program easy to use. Older participants and less educated participants found the sound (reading the information and questions on each screen) helpful compared with the younger and more educated participants.	Not applicable.
Gutierrez et al [36]	Mean age 65.2 years	Patients from a VA^a^ hospital in rural Wisconsin	Mixed methods	Telehospitalist	Communication benefits Telehospitalists reported confidence that the diagnosis accuracy and quality were that of in-person. Patient satisfaction showed improvement in care coordination (18%; P=.02).	Connectivity problems were prevalent, although most providers were able to resort to a backup plan. Internet connectivity was inconsistent, leading to disruption in video communications. It was easy to contact bedside providers/telehospitalist: 100% telehospitalist, 42.9% physicians, 10% nurses, and 22.2% other staff.	Not applicable.
Hatch et al [37]	47.4% of the sample is 65 years or older	33.1% of the sample resided in a rural area	Descriptive cross-sectional cohort	Telehealth	Not applicable.	Older and low-education patients are less familiar with the required technology and experience some technological limitations.	Not applicable.
Hicks et al [38]	Mean age 68.9 years	Residents in rural midwestern state	Experimental	Telemonitoring	78.3% indicated that the use of telehealth technology improved their care. 78.3% indicated it was very convenient. 95.7% of the respondents indicated that the telehealth technology affected their relationship with their nurses positively.	95.7% indicated it was very easy to communicate with the agency personnel using the equipment. 95.7% of the respondents indicated that the telehealth technology was very easy to use. 87.0% indicated that the telehealth technology worked very well.	91.3% of the respondents indicated definitely and 8.7% indicated maybe when asked if they would use the digital health technology again.
Holloway et al [39]	Mean age 61.3 (SD 11.6) years	Residents in rural Montana	Pretest-posttest	PRISM^b^ digital health care videoconferencing	Patients learned to work with the technology, enabling them to interact effectively with patients at other sites. Participants felt that the ability to participate in a team approach to diabetes management without traveling was beneficial. Compared with baseline, patients reported improvements in diabetes care of 30-200% 1 year after the intervention.	Scheduling was a difficult and time-consuming task. Staff members easily learned to use telehealth technology. In most cases, patients were able to learn manual skills using telehealth technology. 99% felt the technology picture and sound were clear. 100% of patients felt comfortable learning health information using this technology and said they understood the information as if it were imparted in person.	Not applicable.
Khairat et al [40]	Mean age 77.8 years	66.7% of the sample resided in rural North Carolina	Cross-sectional	Videoconferencing follow-up care	Geriatric patients did not have trouble using digital health tools. Telemedicine platforms improve primary care by allowing providers to follow-up with their geriatric patients at a time and place that is most convenient for both groups.	Not applicable.	Not applicable.
Kulcsar et al [41]	Mean age 60.3 (SD 16.1) years	The majority of patients resided in rural New Hampshire or rural Vermont	Cross-sectional quality improvement	Telerheumatology	Providers reported that 19% of the patient visits seen via Telerheumatology were inappropriate for the telemedicine visit type due to poor understanding of symptoms, symptom complexity, and the limited ability to perform a physical examination. 81% of patients rated that they were comfortable with the provider’s ability to examine them, thought the provider spent an adequate amount of time with them, and made an accurate diagnosis of their condition.	94% of patients felt that each individual member of the staff made check-in easy, was friendly, and competent with the videoconferencing equipment. An area of greatest dissatisfaction for patients stemmed from problems with scheduling appointments (usually follow-up).	About half (53%) of the patients either agreed or strongly agreed that they would like to be seen via telerheumatology again if given the option.
Liu et al [42]	Mean age 67 years	Patients from a clinic in rural Wisconsin	Qualitative	Teleophthalmology	Teleophthalmology was often preferred by patients because it was more comfortable than traditional eye examinations, since pharmacological pupil dilation was usually not needed. Teleophthalmology is effective for increasing diabetic eye screening rates in rural populations.	Tele-ophthalmology offered convenience through same-day scheduling, proximity to the patient’s primary care provider, and short wait times. Patients appreciated the quick, easy, and painless nature of tele-ophthalmology compared with traditional, in-person eye examinations.	Participants reported being unaware of tele-ophthalmology prior to the study.
Locke et al [43]	Mean age 69.2 years	Residents of rural zip codes defined by the United States Census Bureau	Retrospective	Home computer video health technology	96% of participants preferred video telehealth rather than traditional training visits at the medical center. 76% of participants would not have gotten additional inhaler training if not for the telehealth. The telehealth inhaler training delivered via internet video telehealth demonstrated an improvement in technique overall. The main benefits, as listed by participants of the program, were convenience, time-saving, and decreased travel expenses. Inhaler training delivered via video telehealth by a pharmacist was well received. The CHAT inhaler training program provided an alternative to in-person visits for rural patients with transportation burdens.	Among 93 home telehealth program enrollees, 19 (20%) faced technical issues with the computer or video software that prevented participation. A quarter of participants reported frequent technical problems, consistent with pharmacists noting issues in 149 (63%) of scheduled visits, and 19 visits (13%) were postponed or partially completed due to unresolved technical issues. Common issues included patient errors or confusion (41% of visits) with the video telehealth program, particularly with logging into the Jabber program and basic computer skills, along with 11% experiencing computer/software issues and 25% having audio/video troubles. Despite these challenges, over 90% of participants found the equipment easy to set up and appreciated the benefits of convenience, decreased travel time and expenses, and increased privacy.	A majority (96%) preferred home video telehealth for inhaler training compared with going to the medical center for in-person training.
McIlhenny et al [44]	Mean age 63.8 (SD 12.46) years	Patients of rural medical clinics	Quasi-experimental	Computer-based telemedicine	Not applicable.	Nearly all participants found the information easy to access.	Participants responded positively to the individualized education provided by the nurse educator and did not prefer receiving information from the internet.
Owolo et al [45]	Mean age 59.9 (SD 13.5) years	28.9% of the sample was considered rural	Retrospective cohort	Web-based telemedicine	Patients who used the patient portal had 5.21 times higher odds of completing a video visit compared with patients who did not use the patient portal (95% CI: 1.28-21.23; P=.022). The majority of telehealth visits were conducted over the phone; however, there was an increase in video visits in the post–initial surge period.	81.9% of physicians agreed that telemedicine was easy to use with a preference for imaging review, initial appointments, and postoperative care. While telephone visits were still used at higher rates than video visits, the increased use of video visits potentially reflects a better organized infrastructure for performing this type of visit.	Not applicable.
Robinson et al [46]	Mean age 71 (SD 6.8) years	Residents from rural New York	Cross-sectional	Telemedicine	72% found the in-home nurse visit “very helpful,” in contrast to 55% for telephone tutoring and 46% for the 39-page user’s manual. 37.8% of the participants wanted additional telephone training to access the web.	Twenty-two subjects (63%) rated the amount of training as “3” (about right) compared with their initial expectation. Four responders (11%) rated the training lower than “3” (less than expected), while 9 participants (26%) rated it higher than “3” (more than expected).	Not applicable.
Rodriguez et al [47]	Mean age 63 (SD 12) years	Patients and providers from rural medical centers and remote community-based outreach clinics in Pennsylvania	Mixed methods	Electronic consultations	Improved communication, as it enabled effective information transfer and patient-centered care. For PCPsc, time efficiency was the main reason for telehealth satisfaction. E-consults improved access to specialty care, saving travel time, and enabling confident care.	Not applicable.	All patients indicated that they intended to use e-consults in the future. The intent to use e-consults in the future focused primarily on quality of care and timeliness of care.
Schlittenhardt et al [48]	Mean age 54 years	78% of the sample from rural areas	Mixed methods	Tele-Continence care	The option of telehealth as a follow-up appointment reduced the overall failure rate from 45% to 14.3%. Patients appreciated the convenience and reduction in transportation costs.	Tele-Continence Care implementation was uncomplicated.	When asked if face-to-face visits would be preferred over telehealth visits, patients described a neutral opinion.
Schooley et al [49]	Mean age 63.5 years	Residents of rural Vermont	Mixed methods	Information technology mediums	Telehealth programs, such as telephone triage, are accepted as an option to receive remote care and avoid travel. The community care home telehealth enabled physiological data to be monitored remotely through landline phones amid staffing problems. Email, computers, and assistive devices were imperative to aiding veterans with disabling conditions to communicate effectively while remote.	Assessing mental health symptoms was challenging through telephone and email, as these methods do not allow for observing nonverbal cues, unlike advanced videoconferencing.	56.5% of respondents reported some level of likelihood of consulting with a doctor over the phone, compared with 13.2% who reported some level of likelihood of using the Internet to consult with a doctor. 35% had an interest in communicating with their doctors and nurses via the Internet about their health care. Younger veterans (age 41-55 years) were more likely than older veterans to report interest in the program.
Silvestrini et al [50]	Mean age 60 years	Patients referred from clinics in rural Washington, Oregon, or Alaska	Qualitative	TelePain	TelePain reduced transportation barriers and travel costs. Patients enjoy the convenience of its general accommodations.	Patients generally did not have significant issues using telehealth technology, with many veterans rating TelePain video and audio quality as “good” or “fine.” TelePain staff effectively helped veterans navigate any technological problems, such as resolving audio issues by having the provider call the patient. Some patients suggested improvements for telehealth, such as larger TVs, better monitors, or improved camera systems to enhance the video quality and overall experience.	Many of these patients mentioned that they would like to continue to use TelePain because of its convenience, rather than having to travel far distances to receive pain care. When asked if they would use TelePain again, one patient said, “Oh, of course. It’s much easier than going clear to [the VA medical center].” Another patient replied, “Yeah, probably. It’s easier for me than driving to [the VA] and back.”
Strowd et al [51]	Mean age 44.5 (SD 24.1) years	26% of the sample resided in a rural zip code	Retrospective cohort	Video and phone-based digital technology	73% of respondents reported that clinical needs were met with the telehealth visit. Patients completing the video visits were more likely to have needs met than those who did telephone-only visits (77% vs 71%, P=.34). This was further seen in both urban and rural communities (71% vs 80%, P=.27).	The most common patient-reported barrier to scheduling a video visit was patient technology-related (44% of patients), which included lack or limited access to a smartphone or home computer (n=59), no camera for video (n=51), no internet availability (n=27), or other (n=12).	45% of the respondents would definitely consider a future telehealth visit, 28% might consider, 24% would only consider if required. Patients who completed video visits were more likely to definitely consider a future telehealth visit compared with patients who completed a telephone-only visit (58% vs 38%, P=.02). Patients from rural communities were more likely to definitely consider a future telehealth visit compared with those from urban communities (55% vs 42%, P=.05).
Svistova et al [17]	A large portion of the sample is aged 65 years and older	Rural residents in Pennsylvania	Qualitative focus group	Zoom teleconferencing	Technology has allowed service providers to adapt quickly to COVID-19. The effects of telehealth eliminated the transportation barrier and reduced cancellations and missed appointments.	Older adults faced significant barriers using telehealth due to unfamiliarity with technology, lack of recent devices, low comfort levels, and distrust leading providers to have fewer positive insights about serving them during COVID-19. An insurance provider noted that older clients often lack the proper technology or skills for telehealth. Using technology for health care communication is often uncomfortable for older adults.	Not applicable.
Switzer et al [52]	Mean age 70 years	Patients from one of 12 hospitals, 10 of which were in rural Georgia	Cohort	Telestroke	Telestroke helped enroll patients into the acute stroke treatment program. The initiation and treatment period for Telestroke patients was quicker compared with in-person patients.	Not applicable.	Not applicable.
Virmani et al [53]	Mean age 65.8 (SD 9.2) years	Residents in rural Arkansas	Cross-sectional	Web-based televideo	82% of participants reported televideo had a positive effect on their in-home visits. 70% of participants reported that they liked that there were no travel arrangements. 84% of participants reported that they liked the ability to stay in the comfort of their home. In rural areas, the quality of the audio-video connectivity was enough to implement the routine clinical and research assessments.	28% preferred for in-person visits. Audio-video quality was rated great for 60% of visits, with video slightly slow for 30%. Only 14% of participants needed more than 5 minutes for setup; 42% needed additional time for understanding, locating, or completing research assessments.	Most patients reported being more likely to participate in telemedicine research in the future. 28% preferred for in-person visits.
Waymouth et al [54]	Providers of older adults	Providers in a rural area	Qualitative	Assistive and remote monitoring technology	Telehealth helped facilitate care for individuals with dementia through the use of assistive technology and remote monitoring technology such as fall sensors, webcams, alarm systems, GPS tracking, and smart home hubs.	Not applicable.	Not applicable.
Weiner et al [55]	Mean age 69.7 (SD 12.8) years	Residents of Choctaw Nation of Oklahoma	Clinical trial	Videoconferencing	Virtual care was used to overcome transportation burdens.	Less than 3% no-show rate indicates the technology was easy to use.	There were no refusals of videoconferencing for initial visits and 2 refusals of continued videoconferencing follow-up. The no-show rate for all videoconferencing sessions in the past year was 3%.
West and Milio [56]	Mean age 68.3 years	Patients from a rural homecare organization	Mixed methods case study	Rural homecare organization telemedicine	There were frequent issues with the telehealth device that caused disruptions. Restrictions were seen in outdated telecommunications equipment, which impaired the transmission of audio and visual information. An increase in productivity and lower costs needs to be implemented to meet the demand for these services.	Informants reported that the complexity of the setup and the number of components confused and intimidated the nurses and patients. Faulty equipment also caused frequent disruptions during telemedicine visits.	Younger patients are more likely to receive telemedicine visits than those receiving traditional home care.
West et al [57]	55 years or older	Residents of rural upstate New York	Cohort clinical trial	Web-based telemedicine with videoconferencing	The IDEATel project demonstrated the home televisits through videoconferencing were possible for rural underserved elderly adults with the right home education and behavior goal setting.	Many participants initially had difficulty using the unit and required additional instruction.	Not applicable.
Zulman et al [58]	Mean age 56 (SD 17) years	53% of the sample as rural	Mixed methods	Tablet-based health technology	The VA’s use of video telehealth tablets reached rural older adults effectively.	Not applicable.	81% of tablet recipients used their tablets during the evaluation period. Tablet recipients were more likely to use their tablets if they were 45-64 years or 65 years old (compared with < 45 years). Patients who did not use the telehealth tablet were largely younger with more chronic conditions and a lack of social support.

^a^VA: Veterans Health Administration.

^b^PRISM: Promoting Realistic Individual Self Management.

^c^PCP: primary care provider.

### Quality Appraisal

The quality assessments were achieved through critical appraisal tools used in Joanna Briggs Institute Systematic Reviews [59] and the McGill Mixed Methods Appraisal Tool Version 2018 [60]. Two reviewers assessed the quality of each included article represented in the extracted data. Weekly meetings were held to discuss conflicts. Studies with a quality score of less than 50% were not included in the analysis, indicating evidence of reporting bias.

## Results

### Overview of Reviewed Studies

**Figure 1:**
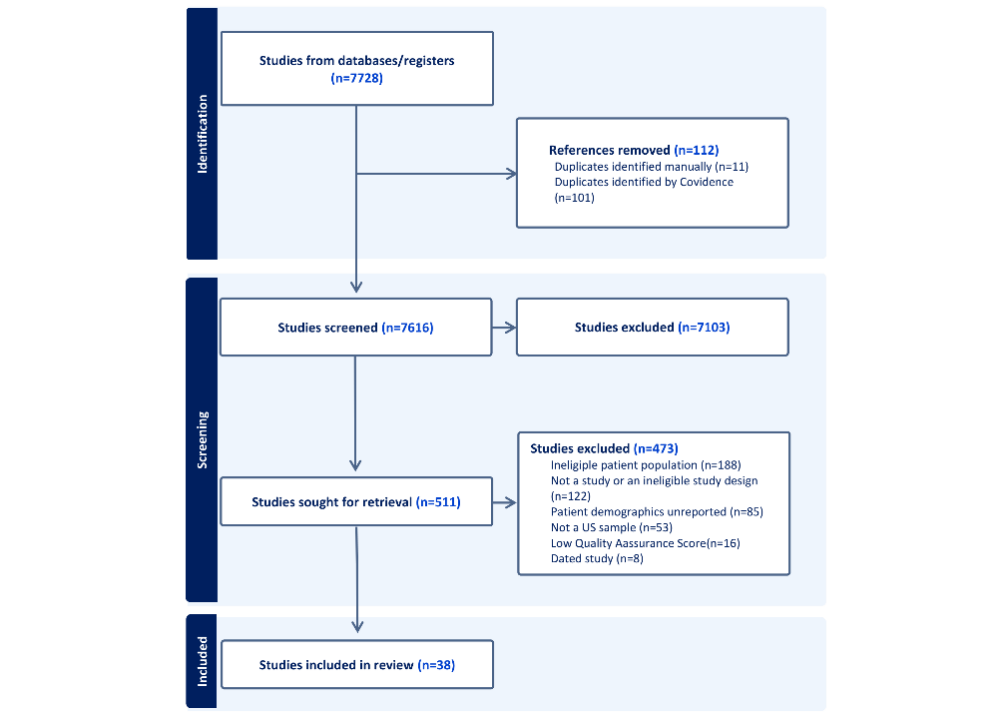
PRISMA flow diagram.

Figure 1 provides a summary of the selection process and illustrates our systematic review of the literature. The preliminary title review produced 7728 results, out of which 7616 underwent the abstract review, and 511 completed the full-text review. Following the assessment, 16 studies were excluded because they scored less than 50% of the total possible score for each respective quality test. Our final review included 39 studies.

Table 1 details our data extraction, elucidating article information according to the first author and publication date, study population age, study design, technology used, and TAM domains. Since 25% of the population had to be at least 60 years old for eligibility, it is important to highlight that studies reported the mean age, included samples exclusively of older adults (eg, Medicare enrollees), and provided the percentage of participants within specific age ranges. The study design of eligible studies was cross-sectional (n=8), qualitative (n=7), and mixed methods (n=11). We will detail lessons learned from the eligible studies according to the TAM domains below.

### Technology Usefulness

Digital health tools were useful for connecting patients to providers [25,26,35,38,54,61], assisting participants with improving health outcomes and care management [25,31,38-43,47-49,51-54,57,61], and reducing transportation burdens [17,23,30,34,43,47-50,53,55]. Uniquely, Anderson et al [23] reported on the role of telehealth videoconferencing in strengthening patients’ social and peer connections. Providers also considered that digital health tools were useful for care management [24,29,32,36,40,49] and communicating with other providers [30,32,47]. Although the respective technology was considered useful, multiple studies reported that rural older adults needed additional supports to facilitate the usefulness of the technology, including behavioral goal setting [57] and assistive devices [49].

Despite the reported usefulness of digital health tools, some studies also reported that they were less than useful. Bernacchi et al [25] revealed that digital health technology was less useful for participants with limited experience with digital technology. Similarly, according to Kulcsar et al [41], nearly one in 5 patients scheduled for a telerheumatology visit was deemed unsuitable due to poor symptom understanding, symptom complexity, and the limitations of conducting a physical examination remotely.

### Ease of Use

Articles that addressed the digital health tools’ ease of use described the tools as uncomplicated [48], requiring an adequate amount of training to use [62], easy to access health information [39,44], easier than an in-person examination [42], easy to communicate with a health care professional [36,38,61], and intuitive [26]. Hybrid models that provided supplemental real-time instruction with the technology improved the ease of use [41,50]. Importantly, some digital health tools were easier to use than others. For example, Schooley et al [49] assessed that mental health evaluations via email and telephone were challenging because of barriers to observing nonverbal cues, yet were easier to accomplish via a videoconference. Additionally, Geller et al [35] reported that audio features improved the ease of using a computer-based intervention for older adults. Providers also reported that digital health tools improved the ease of managing appointments [24,45], accessing and reviewing patient records [24,45], and contacting patients [26]. Weiner et al [55] reported that a low “no-show” rate (3%) evidenced the ease of using videoconferencing visits. Locke et al [43] reported that 90% of the providers reported that the technology was easy to use, despite the fact that more than half of the providers reported issues with scheduling, and 41% reported that patients were confused about using the technology.

Commonly, studies have reported on the challenges of using technology. Digital health tools were more complicated to use when faced with environmental barriers (eg, poor lighting) [23,28], technical or equipment issues [17,25,30-32,36,43,51,53,56], discomfort with the technology [17,25,30,56], reduced access to adequate internet [30,32,36,51], and distrust in the technology [17]. DeHart et al [30] and Hatch et al [37] observed a positive relationship between education levels and ease of use, which was particularly challenging for older adults with low levels of education. Although many studies reported the challenges of using digital tool technology, West et al [57] reported that supplemental instructions were critical for overcoming usage barriers.

### Intention to Use the Technology

Most commonly, participants reported an intent to use the digital health tool technology through sustained use poststudy [34,38,41,47,51,53], future referrals or recommendations [28], high levels of digital tool uptake [43,50,55,58], and evidence of improved care management [25,43,47,50]. For example, Locke et al [43] reported that 96% of the participants preferred home video telehealth inhaler training rather than going to the clinic for in-person training. Bernacchi et al [25] detailed another example of intention to use videoconferencing technology through the patient’s commitment to contact their health care provider despite challenges with equipment and broadband. Strowd et al [51] and Finley et al [34] reported that rural residents were more likely to consider telehealth in the future compared with urban residents. A study indicated that participants were more likely to continue with specific delivery modes of digital health tools, such as telephones, rather than web-based options [49].

When participants did not intend to use the digital health tool technology, it was often due to a preference for in-person visits [24,34,44,45,53,56] or a lack of buy-in about the technology [30,42]. Additionally, 2 studies identified age associations with intentions to use digital health tools. Older adults had reduced intentions of continuing their care digitally [49,51].

## Discussion

### Principal Findings

This review aimed to assess lessons learned about the acceptability of digital health tools among rural older adults. Following a systematic review approach, we organized our findings according to the TAM, focusing on the usefulness, ease of use, and intention to use digital health tools of rural older adults. The domains of the TAM aim to detail predictors of potential acceptance or rejection of the technology. Our findings revealed that digital health tools were, in most cases, useful for care management, reducing transportation burdens, and improving patient-provider communication. Two articles reported that digital health tools were less useful when the technology was misaligned with the participant’s digital skills level or when the technology was unsuitable for the given care visit types. Several articles highlighted the ease of use of digital health tools for rural older adults, describing them as uncomplicated, intuitive, and effective for connecting with health care providers. Most articles that discussed the ease of using digital health tools focused on the ease of specific features of the tool (eg, audio capabilities) or the type of technology (eg, telephone vs digital). However, digital health tools were more difficult to use due to technical or equipment issues, discomfort with the technology, and limited access to broadband internet. Last, the findings on rural older adults’ intention to use digital health tools were robust, as evidenced by participants’ preference to continue using the technology after the study concluded and improved health outcomes. Comparatively, participants did not intend to use the digital health tools when they preferred an in-person visit or when they were not sold on the benefits of the digital health tool. Together, the TAM domains reveal that rural older adults and their providers largely consider digital health tools as acceptable modes of receiving care and, at times, a suitable alternative to in-person clinic visits. Despite barriers that reduced rural older adults’ acceptance of digital health tools, many of these barriers were not associated with their age or rural residence.

### Technology Usefulness: Lessons Learned

Digital health tools are useful for accessing health care and care management, but their effectiveness in improving health outcomes in older adults is mixed. Our review highlights that digital health tools were useful for mitigating burdens related to accessing health care and care management for both providers and patients. Articles reported that useful care management needs included scheduling, accessing health records, and patient-provider communication. The positive findings on remote care management and usefulness are specific to this review, and it is important to note that the effectiveness of remote care management and monitoring on health outcomes is mixed. In a review of remote care management of depression and anxiety in older adults, the findings on psychiatric outcomes were mixed, and no studies demonstrated a statistically significant effect of remote care management on health care use or cost [63]. Another review of mobile integrated health interventions for older adults revealed that these interventions reduced emergency department call volume and transports [64]. Thus signaling that digital health tools were useful for care management during emergency health events.

To build on this body of evidence, our review uniquely emphasizes the usefulness of digital/remote care management for rural older adults—a population that has complex care needs but often resides in a medically underserved area with reduced access to broadband internet and technology literacy programming. However, future systematic and meta-reviews are needed to assess the effectiveness of digital health tools with care management features on health outcomes and costs for rural older adults.

### Ease of Use: Lessons Learned

Easy-to-use technology is associated with improved health outcomes; however, the design of technology may not be sufficient, and external support (eg, timely technical assistance) may be necessary. According to the TAM, digital tools that are perceived to be easy to use are more likely to be accepted by the intended audience. Based on this review, rural older adults and their providers frequently highlighted user-friendly features of the tools that improved ease of use. However, there was strong evidence that external factors—such as technical issues, equipment limitations, and discomfort with the technology—hindered usability. There is a positive relationship between the health of older adults, their social connectedness, access to high-quality health care resources, and the perceived ease of use of digital health tools [46,65]. Notably, this evidence signals that technology design alone does not improve the ease of using digital health tools. Specifically for older adults, in-person synchronous technical assistance, access to remote technical assistance, and early interventions from hospital administrators are reported facilitators for increasing the ease of use of digital health tools [15,25,29]. Overall, this evidence underscores the need for additional resources and external support to enhance the perceived ease of using digital health tools for rural older adults.

### Intention to Use the Technology: Lessons Learned

Despite design flaws or technical difficulties, rural older adults generally intended to continue using technology beyond the observed period. Factors such as the preference for in-person care and a lack of buy-in about the technology influenced the participants’ intent to use digital health tools. Yet, our findings revealed that participants described an intention to use digital health tools despite also reporting that the technology was not always useful or easy to use [25,34,41,43]. In a similar study on patient portal use by older adults, challenges were noted with log-ins and the user interface design, such as color and font [62]. Despite these issues, older adults expressed an intention to continue using the portals due to their other beneficial features. In summary, in light of the barriers to ease of use and usefulness, rural older adults often overcame them to continue using digital health tools [25].

### Additional Considerations for Digital Tool Acceptability

In synthesizing lessons learned, our review identified several phenomena, not salient enough to categorize as a lesson, but worthy of continued discussion. Namely, our review reveals both differences and commonalities in user behavior and preferences between older adults in rural and urban areas. As reduced access to broadband internet is a common barrier for rural residents, this was not the most reported impediment to digital tool acceptability in this review, as expected. According to the Federal Communications Commission’s data reported in 2021, 23%-50% of rural residents had poor access to broadband internet [66]. The rather limited mention of challenges associated with rural internet connectivity, in this review, is inconsistent with the existing literature, which indicates that poor internet connection is a key barrier to digital tool use acceptability for rural residents [15]. Also notable is that multiple studies reported a measure of digital tool acceptance among rural residents compared with urban residents. For example, Finley et al [34] reported urban-rural differences in intention to use, indicating that rural patients, compared with urban and suburban patients, had more favorable attitudes toward telecardiology. This higher acceptance of digital health tools by rural patients likely punctuates the growing reliance on and acceptance of digital health tools in the wake of dwindling local health care resources and rising health care costs. While these findings suggest that rural and urban residents share common barriers to technology acceptability, our conclusions do not suggest that “one-size-fits-all” interventions should be considered. Rather, additional qualitative examinations on the rural-urban differences in attitudes toward digital health tools and the acceptability of these tools are warranted.

This review synthesized a diverse representation of technology modes (eg, videoconference or phone call), highlighting the robust intervention designs implemented in rural settings. These findings strengthen the evidence base on digital tool acceptability among rural patients, with all modes being reported as acceptable. Yet, important distinctions emerged across the reported technology modes, making salient conclusions about the most acceptable modes speculative. For example, both Anderson et al [23] and Svistova et al [17] used videoconferencing; however, Anderson et al [23] employed a Veterans Affairs-supported platform, and Svistova et al [17] used Zoom. The journal articles provided limited details regarding platform-distinctive features, though such distinctions could plausibly impact acceptability and usability very differently. In the current review, technology modes were extracted as they were identified in the original article to ensure transparency. A more detailed analysis of the technology’s distinctive features and their impact on acceptability warrants further investigation.

The data collected for this study includes both pre– and post–COVID-19 pandemic publications, which provide key insights into how rural older adults’ acceptance of digital health tools evolved, resulting from the rapid uptake of telehealth due to COVID-19 precautions. Many of the studies that were published prior to the COVID-19 pandemic often emphasize the same sentiment of the digital divide between younger and older generations. The existing literature evidences that the pandemic exacerbated this divide, as prepandemic older adults were less likely to benefit from technological innovations [67]. Specifically, pre- and early-pandemic trends indicated that age and rural zip codes were inversely related to continuous digital tool use [68]. Multiple studies from our review that were published prepandemic identified that younger participants were more likely to engage in digital tool technologies [49,56]. Yet, in a peripandemic investigation, Bernacchi et al [25] describe that videoconferencing with a nurse increased access to care for older rural cancer patients. Similarly, postpandemic, individuals older than 65 years used telemedicine over 3 times more when compared with prepandemic, further emphasizing this technological shift [19].

The findings of this review have practice and research implications. To improve practice, our findings suggest that health care providers adopt hybrid care models—combining both digital and in-person visits. It is essential for providers to emphasize the benefits of digital health tools, such as reducing travel burdens and offering greater convenience [30,39,43,47,49,50,53]. Additionally, future research should include interventions aimed at increasing technology literacy among rural older adults and provide additional synchronous and asynchronous supports to help rural older adults better understand the technology [17,25,30-32,36,37,51,56,57]. For caregivers who support rural older adults, digital health tools have the potential to reduce caregiving burdens. Tools such as remote monitoring enable doctors and nurses to keep patient health under observation while the patient remains in the comfort of their home [30,38,54]. This technology could reduce the burden on caregivers and limit accidents. Last, it is our hope that these findings will influence policy that increases funding for the development of digital health tools that can decrease health disparities within rural older adult populations.

### Limitations

This study has several strengths and limitations. The synthesis of our findings was guided by the core domains of the TAM [69]. Previous studies have reported that the Perceived Usefulness, Perceived Ease of Use, and Intention to Use domains are important for determining user acceptability. They are the core of all TAM models used across the literature, thus chosen for this study. Yet, additional domains and extensions have been added over time, including the “Attitudes Toward Using” domain [70,71]. Although our synthesis is limited to conclusions derived from the core domains, these domains provide sufficient information to inform our overall aim of assessing user acceptability. Additionally, a trained librarian conducted a data search of publicly available databases (eg, PubMed). Despite our comprehensive search conducted by a trained librarian, some relevant studies may have been missed due to factors such as publication dates postreview, non–English language restrictions, or potential oversight in keyword selection. It is important to note that participants in health outcomes research are often younger than 65, have higher household incomes, greater technological literacy, and are more likely to reside in urban areas [27,33,72]. Therefore, our review’s focus on rural older adults and digital tool use may highlight a potential participant bias in our sample, limiting the generalizability of our findings to the broader rural older adult population. As with all scholarly reviews, researcher bias could have impacted the results. However, because multiple reviewers assessed each manuscript and attended consensus meetings for each manuscript, this bias was reduced.

### Conclusion

This study aimed to systematically review articles that incorporated digital health tools used by rural older adults in order to assess their acceptability and usage of the tools. Following the TAM, we highlighted the usefulness, ease of use, and intention to use digital health tools. In summary, digital health tools were valuable for rural older adults with complex care needs, helping mitigate access barriers and support care management tasks like scheduling and patient-provider communication. While rural older adults and providers found the tools user-friendly, external factors such as technical issues and equipment limitations impeded usability, signaling the need for additional support and resources. Despite challenges with ease of use, rural older adults expressed an intention to continue using digital health tools, recognizing their overall benefits in managing care, especially in underserved areas. As medical deserts widen in rural communities, and in response to the rapid uptake of telemedicine due to COVID-19 precautions, digital health tool reliance is likely to grow for rural residents. Understanding what this uniquely vulnerable population views as acceptable and what facilitates their uptake of digital health tools is critical for addressing health disparities and bridging the digital divide in rural communities.

## Appendix

Multimedia Appendix 1 PRISMA checklist.

## Abbreviations

PRISMA: Preferred Reporting Items for Systematic Reviews and Meta-Analyses.
TAM: technology acceptance model
